# Genome-wide characterization of major latex protein gene family in peanut and expression analyses under drought and waterlogging stress

**DOI:** 10.3389/fpls.2023.1152824

**Published:** 2023-04-18

**Authors:** Jie Li, Ruier Zeng, Zijun Huang, Hengkuan Gao, Shiyuan Liu, Yu Gao, Suzhe Yao, Ying Wang, Hui Zhang, Lei Zhang, Tingting Chen

**Affiliations:** Guangdong Key Laboratory of Plant Molecular Breeding, College of Agriculture, South China Agricultural University, Guangzhou, China

**Keywords:** peanut, major latex protein, phylogenetic relationship, gene expression, abiotic stress

## Abstract

Peanut is an important oilseed crop around the world which provides vegetable oil, protein and vitamins for humans. Major latex-like proteins (MLPs) play important roles in plant growth and development, as well as responses to biotic and abiotic stresses. However, their biological function in peanut is still unclear. In this study, a genome-wide identification of *MLP* genes in cultivated peanut and two diploid ancestor species was analyzed to determine their molecular evolutionary characteristics and the expression profile under drought and waterlogging stress conditions. Firstly, a total of 135 *MLP* genes were identified from the genome of tetraploid peanut (*Arachis hypogaea*) and two diploid species *Arachis. duranensis* and *Arachis. ipaensis*. Then, phylogenetic analysis revealed that MLP proteins were divided into five different evolutionary groups. These genes were distributed unevenly at the ends of chromosomes 3, 5, 7, 8, 9 and 10 in three *Arachis* species. The evolution of *MLP* gene family in peanut was conserved and led by tandem and segmental duplication. The prediction analysis of cis-acting elements showed that the promoter region of peanut *MLP* genes contained different proportions of transcription factors, plant hormones-responsive elements and so on. The expression pattern analysis showed that they were differentially expressed under waterlogging and drought stress. These results of this study provide a foundation for further research on the function of the important *MLP* genes in peanut.

## 1 Introduction

Peanut (*Arachis hypogaea* L.), also named groundnut, is an important industrial crop worldwide, which provides vegetable oil, protein, minerals and vitamins for humans. Cultivated peanut is an allotetraploid (AABB, 2n = 4x = 40) thought to be derived from hybridization between the diploids *A. duranensis* (AA, 2n = 2x = 20) and *A. ipaensis* (BB, 2n = 2x = 20) (Bertioli et al., 2016). During the growth process of peanut plants, their yield is challenged by several environmental factors. Drought and waterlogging are two abiotic stress factors that severely threatened our food security (Huang et al., 2020; Zeng et al., 2021). Breeding abiotic stress-resistant cultivars is one of the most efficient methods to reduce the production and quality losses in peanut.

MLP homologs can be divided into three groups: MLPs, Bet v 1s, and pathogenesis-related proteins class 10 (PR-10s), one of 17 members of the PR family (Fujita and Inui, 2021). PR-10s are mostly cytosolic proteins, constitutively expressed in several plant tissues. Their expression is upregulated under abiotic and biotic stress (El-Banna et al., 2010; Gómez-Gómez et al., 2011). Hence, it has been proposed that PR-10 proteins play a more general role in plant development and defense mechanisms. Bet v 1s were the major allergens of birch (*Betula verrucosa*) pollen. They played important role in steroid binding. The ability of Bet v 1 to bind a broad spectrum of plant intrinsic ligands, such as fatty acids, cytokinins, or flavonoids, has led to an involvement in different stages of plant reproduction (Aglas et al., 2020).MLPs have been firstly discovered in the latex of opium poppy (*Papaver somniferum*) (Nessler et al., 1990). To date, *MLP* genes have been identified in many dicots, including *Arabidopsis* (Chen and Dai, 2010), cucumber (Suyama et al., 1999), ginseng (Choi et al., 2015), grape (Fernandes et al., 2013; Zhang et al., 2018), apple (He et al., 2020; Yuan et al., 2020), kiwifruit (D'Avino et al., 2011), melon (Aggelis et al., 1997), soybean (Strömvik et al., 1999) and cotton (Yang et al., 2015). Like Bet v 1s and PR-10s, a common structural feature of MLP proteins is the formation of a hydrophobic cavity that forms the ligand-binding site for transporting hydrophobic compounds, such as steroids (Lytle et al., 2009), long-chain fatty acids (Choi et al., 2015), and organic pollutants (Inui et al., 2013) *via* phloem and xylem vessels in the plants (Li et al., 2013; Carella et al., 2016; Goto et al., 2019).

In recent years, many studies have elucidated two biological functions of MLP proteins in plant growth and development, as well as abiotic stress tolerance. Firstly, MLPs play an important role in plant growth and development (Guo et al., 2011). It has been reported that the MLPs were related to flower development and fruit ripening in peach (*Prunus persica*) (Ruperti et al., 2002) and kiwifruit (*Actinidia deliciosa*) (D'Avino et al., 2011; Chruszcz et al., 2013). Secondly, MLPs play crucial roles in tolerance to abiotic stress and induction of pathogen resistance *via* the plant hormone signaling pathway (Wang et al., 2015; Yang et al., 2015). For instance, the *MLP* genes were detected in the stem phloem sap of cucumber mosaic virus infected plants (Malter and Wolf, 2011). In cotton plants, ectopic overexpression of the cotton (*Gossypium hirsutum*) *GhMLP28* gene in *A. thaliana* enhanced tolerance to salt stress (Chen and Dai, 2010), while overexpression in *Nicotiana tabacum* enhanced resistance to *Verticillium dahliae* infection (Yang et al., 2015). Additionally, it was reported that overexpression of *AtMLP43* improves drought tolerance by mediating abscisic acid (ABA) signal transduction (Wang et al., 2015).

In order to enrich our knowledge of the roles of *MLP* members in peanut, in this study, 135 *MLP* genes were identified from cultivated peanut and its two diploid ancestor species. Phylogenetic analysis, chromosomal location, synteny analysis, gene structure and cis-acting regulatory elements of their upstream regions were comprehensively analyzed. Furthermore, responsiveness to drought and waterlogging stress were further evaluated. Taken together, these results serve as a genome-wide identification and expression analysis of *MLP* family genes in peanut, and it provided a new way to improve the ability of peanut to resist abiotic stress.

## 2 Materials and methods

### 2.1 Identification of *MLP* genes in peanut

Protein sequences of tetraploid peanut variety Tifrunner, and two diploid species *A. duranensis* and *A. ipaensis* were acquired from the peanut database (https://www.peanutbase.org/). The hidden Markov model (HMM) of Bet v 1 domain (accession: PF00407) was downloaded from Pfam database (http://pfam.xfam.org/) (Finn, 2006).

Peanut *MLP* genes were searched in the protein database by HMMER 3.0 software (E-value <1e^−10^). The candidate protein sequences, CDS sequences and conserved domain sequences were extracted using Perl command. The putative protein sequences were aligned with Pfam (http://pfam.xfam.org/), SMART (http://smart.embl-eidelberg.de/), and CDD (https://www.ncbi.nlm.nih.gov/cdd/) database to further confirmed to contained domains. The physical and chemical properties of the peanut MLP proteins, including the number of amino acids, molecular weight (MW), and theoretical isoelectric point (pI) were analyzed using the online tool ExPASy6 (Wilkins et al., 1999).

### 2.2 Phylogenetic analysis of *MLP* genes

The MLP protein sequences of *Arabidopsis thaliana* were downloaded from the NCBI (https://www.ncbi.nlm.nih.gov/). The MLP protein sequences of *Glycine max* and *Medicago truncatula* were downloaded from the Phytozome database (https://Phytozome.jgi.doe.gov/pz/portal.html/). Multiple alignments of MLP proteins from *Arachis hypogaea*, *Arachis duranensis*, *Arachis ipaensis*, *Glycine max*, *Arabidopsis thaliana* and *Medicago truncatula* were conducted using Clustal W with default parameters. A phylogenetic tree was constructed *via* the Neighbor-joining method with 1,000 bootstrap replicates using Molecular Evolutionary Genetics Analysis (MEGA 7.0) software (Kumar et al., 2018).

### 2.3 Gene structure analysis and protein conserved motifs of peanut MLPs

The peanut gene structure annotation file was downloaded from the peanut database (https://www.peanutbase.org/). The conserved motifs of the peanut MLP proteins were identified using Multiple Expectation Maximization for Motif Elicitation (MEME Suite) (Bailey et al., 2006). The above results are visualized using Tbtools (Chen et al., 2018).

### 2.4 Promoter cis-elements analysis of *AhMLPs*

The upstream regions (2000 bp) from the initiation codon (ATG) of *MLP* genes were defined as the putative promoter sequence. The presence of various cis-acting elements of each sequence was identified by submitting to the PlantCARE database (http://bioinformatics.psb.ugent.be/webtools/plantcare/html/). The results were visualized by TBtools (Chen et al., 2018).

### 2.5 Chromosomal localization and synteny analysis of peanut *MLPs*

The chromosome locations of each *MLP* gene were obtained from the gene annotation file in PeanutBase (https://www.peanutbase.org/). Gene location was visualized *via* the online tool MapGene2Chrom v2.0 (http://mg2c.iask.in/mg2c_v2.0/). The Multiple Collinearity Scan toolkit (MCScanx) in Tbtools was used to analyze gene duplication events of *MLP* genes. Tandem and segmental duplication analysis of the *MLP* gene was performed by the Multiple Collinearity Scan toolkit (MCScanx) in TBtools (Chen et al., 2020).

### 2.6 Plant materials and treatments

In the previous studies, different expressional genes were analyzed by RNA-seq under waterlogging and drought stress, respectively (Huang et al., 2020; Zeng et al., 2020). For the waterlogging stress, peanut cultivars Zhongkaihua 1 (ZKH1, waterlogging-resistant) and Huayu 39 (HY39, waterlogging-sensitive) were planted in plastic pots. At the initial flowering stage, the peanut plants were treated with waterlogging for 5 and 10 days, respectively. The plants without any waterlogging treatment were used as the control (Zeng et al., 2020). For the drought stress, the seeds of variety HY39 were sowed into plastic pots. At the initial flowering stage, the plants were treated under drought for 7 and 14 days (Huang et al., 2020). The waterlogging and drought stress were conducted in the greenhouse of South China Agriculture University. The size of the pots was 410 mm × 335 mm × 320 mm (top diameter × bottom diameter × height).Three experimental replicates were conducted in both waterlogging and drought stress. After stress treatments, at the peak flowering stage, the third leaf located on the top (usually called the functional leaf) was collected and rapidly frozen in liquid nitrogen for RNA-seq and quantitative real-time PCR (qRT-PCR).

In this study, to analyze expression pattern of *MLP* genes in different tissues, the roots, stems, leaves, flowers, pegs, and seeds of peanut variety HY39 were collected and then stored in a refrigerator at -80 °C for subsequent RNA extraction and qRT-PCR.

### 2.7 Expression patterns of *MLP* genes

In this study, to study the expression patterns of peanut *MLP* genes under waterlogging and drought stresses, different expressional genes were analyzed according to project accession PRJNA629848, SRP259445 (for waterlogging stress) (Zeng et al., 2021) and PRJNA629665 (for drought stress) (Huang et al., 2020) from NCBI (https://www.ncbi.nlm.nih.gov/).Heatmaps were generated by Omicsmart website (https://www.omicsmart.com/home.html/), and shows the normalized value Z-score after log2 (FPKM) transformation (Clevenger et al., 2016). Based on heatmap results, four and five candidate genes for waterlogging and drought stress were selected to verify the RNA-seq data, respectively.

The total RNA was extracted following the method specified in the RNA extraction kit (Takara, Japan). The equivalent amount RNA was reverse-transcribed into cDNA using the reverse transcription kit (Vazyme, Nanjing, China). The qRT-PCR was performed using an Applied Biosystems QuantStudio 3 and 5 system (Thermo Fisher Scientific, USA). Using the SuperReal PreMix Plus (SYBR Green) kit, the reaction procedure was as follows: 95°C for 3min, followed by 40 cycles of 95°C for 15s, 57°C for 15s, and 72°C for 20s, 40 cycles. The dissolution curve was analyzed. The primer sequences of the candidate genes are listed in **Table S1**, with *Actin* as the internal reference gene. The relative expression of the target gene was calculated using the ΔCT method. The relative expression of the target gene was 2^− ΔΔCT^, and the experiment was repeated three times.

## 3 Results

### 3.1 Identification and physicochemical properties of MLP proteins in *Arachis*

In order to identify MLP proteins in peanut, the HMM profile of MLP (accession: PF00407) was used to search the peanut released protein database. All the obtained MLP proteins were analyzed by SMART to confirm the whole Bet v 1 motifs. In total, 68, 36 and 31 MLP proteins were obtained from tetraploid *A. hypogaea* (peanut variety Tifrunner, named AhMLP1-AhMLP68), and two diploid species *A. duranensis* (AdMLP1-AdMLP36) and *A. ipaensis* (AiMLP1-AiMLP31), respectively (**Table S2**). The physicochemical properties of the MLP proteins were analyzed online using ExPASy (**Table S2**). The 68 AhMLP proteins contained 64-156 amino acids (aa), with a molecular weight (MW) of 19.62-72.58 kDa. The predicted isoelectric point (pI) ranged from 4.05 to 8.93. Among 36 AdMLP proteins, the length of amino acids ranged from 92 to 309 aa, and the MW ranged from 10.62 to 34.46 kDa. The pI ranged from 5.04 to 9.66. The length of amino acids of AiMLP proteins ranged from 102 to 228 aa, and the MW ranged from 11.66 to 26.35 kDa. The pI of AdMLP proteins ranged from 4.96 to 7.02.

### 3.2 Phylogenetic analysis of the MLP proteins

**Figure 1:**
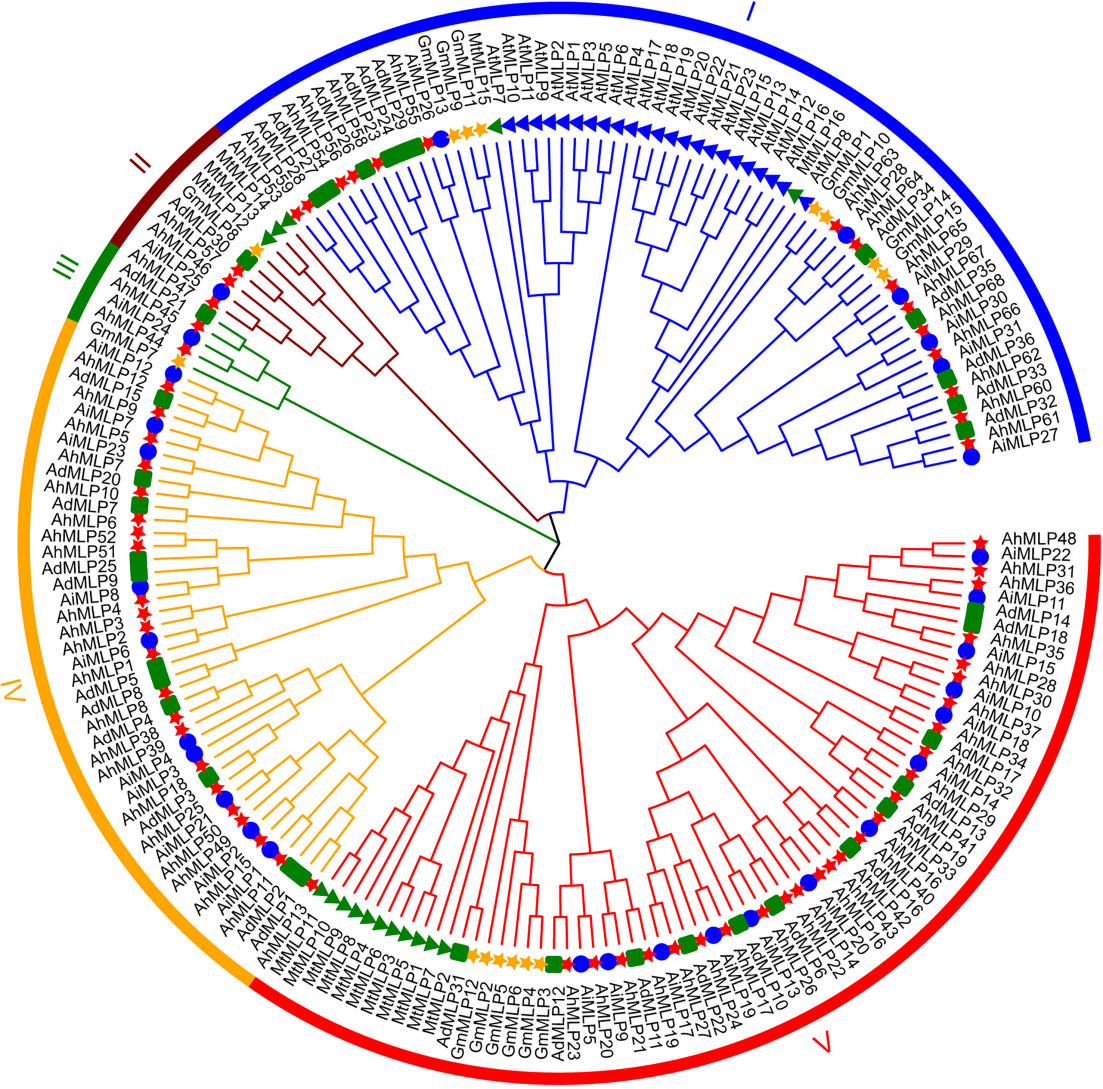
Phylogenetic analysis of MLP proteins in plant. The red star, green square, blue circle, green triangle, blue triangle, and yellow star represent *Arachis hypogaea*, *Arachis duranensis* and *Arachis ipaensis*, *Medicago ruthenica*, *Arabidopsis thaliana* and *Glycine max*, respectively.

To explore the evolutionary relationships of MLP proteins, a phylogenetic analysis was conducted on six species, including *A. hypogaea, A. duranensis*, *A. ipaensis*, *Arabidopsis*, *Glycine max*, and *Medicago truncatula*. A maximum likelihood phylogenetic tree by 1,000 bootstraps was constructed with 68 AhMLPs, 36 AdMLPs, 31 AiMLPs, 23 AtMLPs, 15 GmMLPs and 16 MtMLPs (**Figure 1**, **Table S3**). According to the phylogenetic tree, 189 MLPs from six species were classified into five groups. Group I, II, III, IV and V contained 64, 9, 5, 43 and 68 MLP proteins, respectively. 135 peanut MLPs from three *Arachis* species were randomly distributed among the five groups. 32 (15 AhMLPs, 11 AdMLPs and 6 AiMLPs), 5 (3AhMLPs, 1 AdMLPs and 1 AiMLPs), 4 (2 AhMLPs, 1 AdMLPs and 1 AiMLPs), 43 (22 AhMLPs, 11 AdMLPs and 10 AiMLPs), and 51 (26 AhMLPs, 12 AdMLPs and 13 AiMLPs) MLPs were distributed in Group I-V, respectively. Group IV contained 43 peanut MLPs, without MLPs from other species. Group I contained 32 peanut MLPs, 23 AtMLPs, 7 GmMLPs and 2 MtMLPs. Group II contained 5 peanut MLPs, 1 GmMLPs and 3 MtMLPs. Group III contained 4 peanut MLPs and 1 GmMLPs. Group V contained 51 peanut MLPs, 6 GmMLPs and 11 MtMLPs. It was suggested that peanut is closely related to *Glycine max* and *Medicago truncatula*, compared with *Arabidopsis*. In addition, there are AhMLP, AdMLP, AiMLP in the same clade, which suggested that MLPs from *A. hypogaea, A. duranensis* and *A. ipaensis* were evolutionary conserved.

### 3.3 Chromosomal localization and synteny analysis of *MLP* genes in peanut

**Figure 2:**
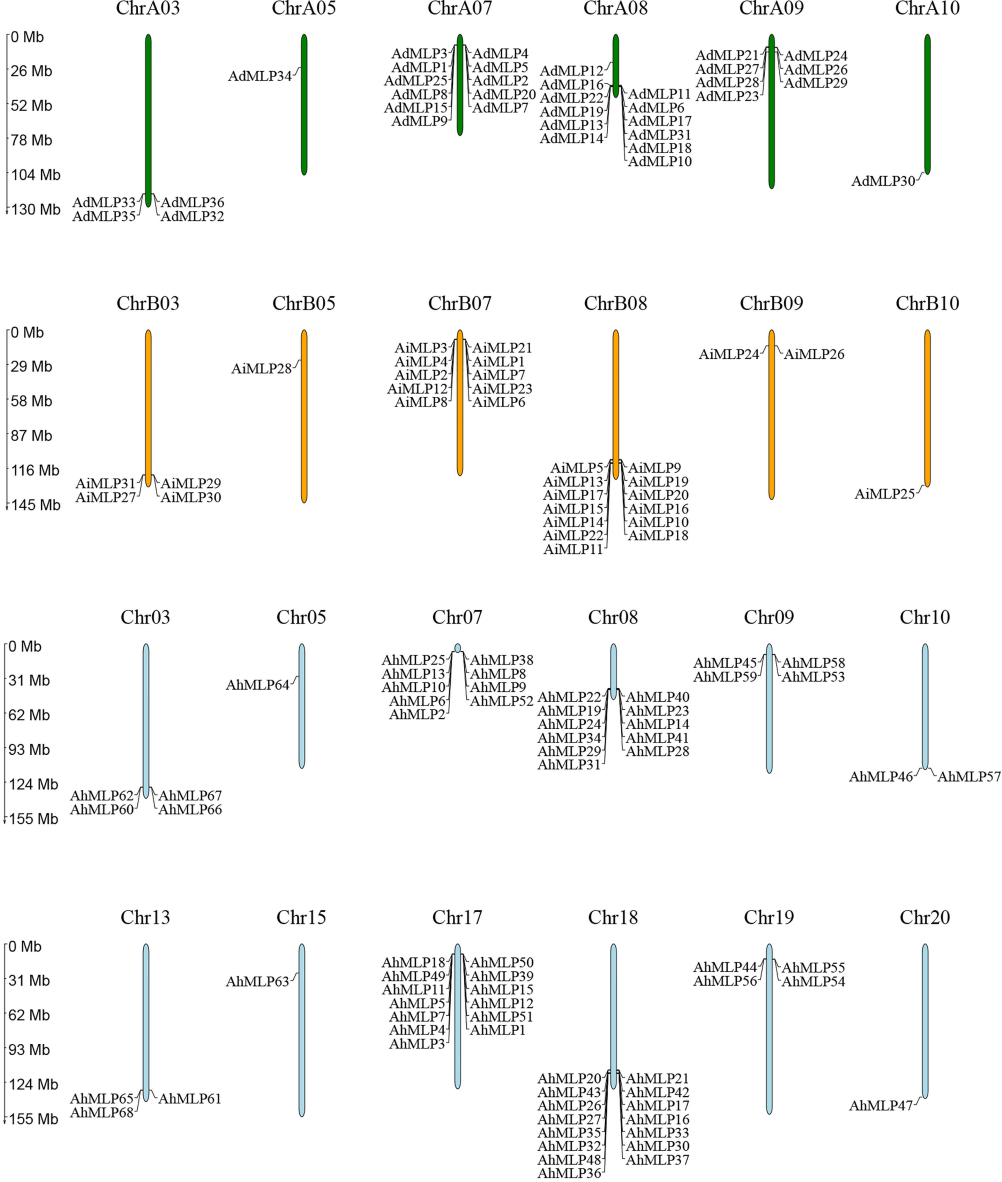
Chromosomal locations of MLPs in Arachis hypogaea, Arachis duranensis and Arachis ipaensis.

**Figure 3:**
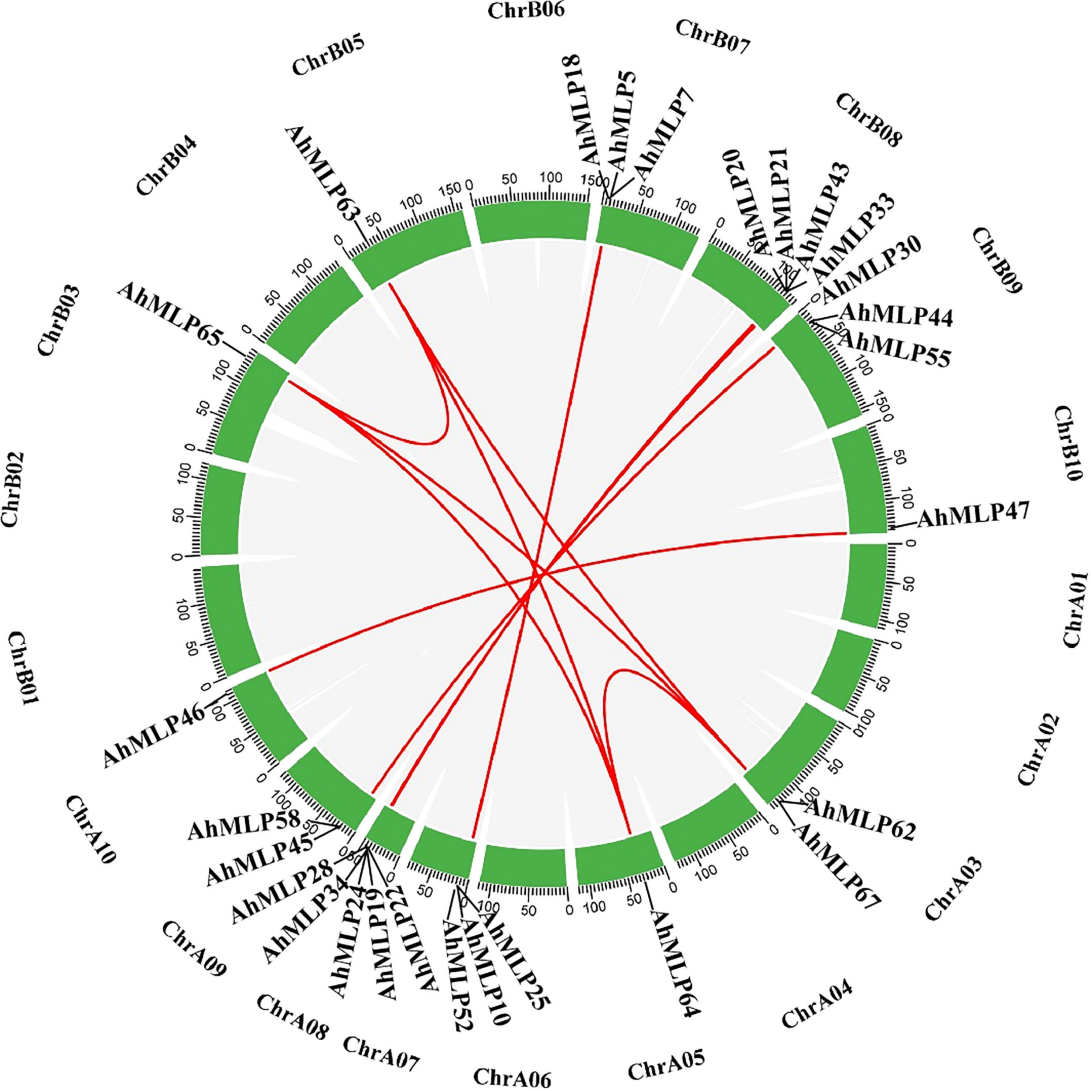
Collinearity analyses of *AhMLP* genes in peanut. Red lines indicate the duplicated *AhMLP* gene pairs in *Arachis hypogaea*.

All 68 *AhMLP* genes were unevenly distributed to 12 chromosomes of *Arachis hypogaea*, including Chr03, Chr05, Chr07, Chr08, Chr09, Chr10, Chr13, Chr15, Chr17, Chr18, Chr19 and Chr20 (**Figure 2**). Mostly, 15 (22.1%), 13 (20.6%), 11 (16.2%) and 9 (13.2%) *AhMLPs* located on Chr 18, Chr 17, Chr 08 and Chr 07, respectively. In total, there were 31 and 37 *AhMLPs* in sub-genomes AA and BB, respectively. Moreover, 36 *AdMLP* genes were located on ChrA03 (4), ChrA05 (1), ChrA07 (11), ChrA08 (12), ChrA09 (7), ChrA10 (1), respevtively. 31 *AiMLP* genes were located on ChrB03 (4), ChrB05 (1), ChrB07 (10), ChrB08 (13), ChrB09 (2) and ChrB10 (1), respectively. In addition, *MLP* genes in peanut were distributed in clusters at the ends of the Chr03, Chr07, Chr08 and Chr09, implying that the members of gene family were possibly led by tandem gene duplication. In addition to tandem duplication, fragment duplication events of *AhMLP* genes family were analyzed. 17 gene pairs with segmental duplications were distributed on 12 different chromosomes (**Figure 3** and **Table S4**). These results suggested that the evolution of *AhMLP* gene family was led by tandem and segmental duplication.

### 3.4 Gene structure and motif analysis of *MLPs* in peanut

**Figure 4:**
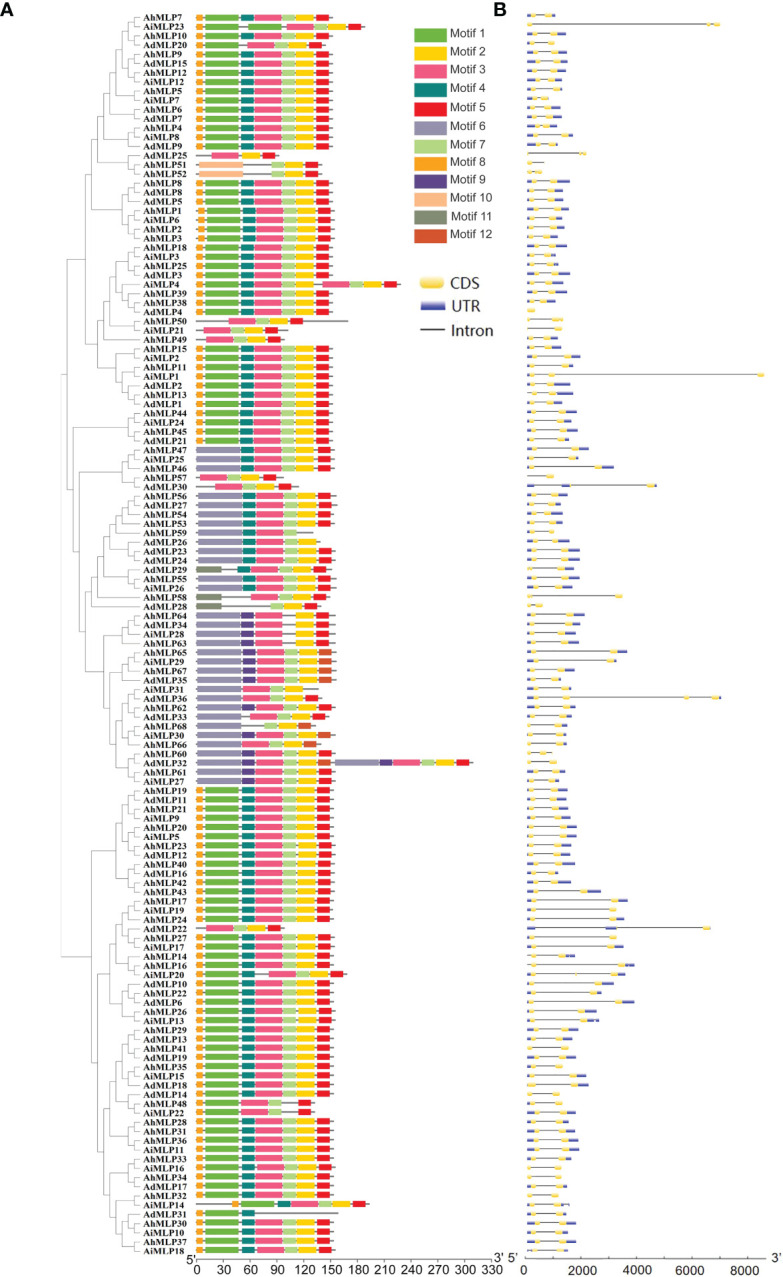
Phylogenetic relationship, conserved motif and gene structure analysis of the *MLP* gene in peanut. **(A)** Phylogenetic tree of the *MLP* gene in peanut and conserved motifs analyses of the *MLP* gene. A total of 12 predicted motifs were showed by different colored boxes. **(B)** Gene structure analyses of the *MLP* gene in peanut.

To further understand the structural characteristics of peanut MLPs and conserved domain, the exon/intron/UTR and protein motif of 135 MLP genes identified in peanut were analyzed (**Figure 4**, **Table S5**). The conserved domains of MLP proteins were analyzed by the online software MEME. 12 conserved motifs (named Motif 1 - Motif 12) were obtained (**Figure 4A**). Phylogenetic analysis showed that 135 peanut MLPs were classed into three groups. Motif 8, 1, 4, 3, 7, 2 and 5 existed in the MLPs of group I, except AdMLP25, AhMLP51 and AhMLP52. In group II, motif 6, 3, 7, 2, and 5 existed in the most of MLPs. Motif 8, 1, 4, 3, 7, 2 and 5 existed in the MLPs of group III, except AdMLP22, AiMLP16 and AhMLP34 (**Figure 4**). The gene structure of *MLPs* in peanut was analyzed by Tbtools (**Figure 4B**). Among them, 114 peanut *MLP* genes contained two exons and one intron. Seven peanut *MLP* genes contained three exons and two introns. Six peanut *MLP* genes contained two exons and two introns. Generally, 97 peanut *MLP* genes contained one 5’ UTR and one 3’ UTR. Besides, 10 peanut *MLP* genes only had one 5’ UTR, without 3’ UTR; Four peanut *MLP* genes had only one 3’ UTR; 14 peanut *MLP* genes had neither 5’ UTR nor 3’ UTR.

### 3.5 Analysis of cis-acting elements in peanut *MLP* genes

To further understand the potential regulatory mechanism of peanut *MLP* gene family in abiotic stress response, the upstream 2000 bp sequence of 135 peanut *MLP* genes with complete domains was extracted from peanut genome information for the analyses of cis-acting elements (**Supplementary Figure S1**, **Table S6**). After removing the components with content ≤ 0.1%, eight cis-acting elements were obtained, including transcription factors of myeloblastosis (MYB) and myelocytomatosis (MYC), ethylene-responsive element (ERE), abscisic acid-responsive element (ABRE), anaerobic induction element (ARE) and so on (**Supplementary Figure S1**, **Table 1**). **Table 1** showed that a total of 130 peanut *MLP* genes contained MYB transcription factors, including *AhMLP* (67, 51.54%), *AdMLP* (35, 26.92%), and *AiMLP* (28, 21.54%). ERE elements existed in 120 peanut *MLP* genes, including *AhMLP* (62, 51.67%), *AdMLP* (30, 25.00%), and *AiMLP* (28, 23.33%). 119 peanut *MLP* genes contained MYC transcription factors, including *AhMLP* (62, 52.10%), *AdMLP* (33, 27.73%), and *AiMLP* (24, 20.17%). ABRE elements existed in 98 peanut *MLP* genes, including *AhMLP* (50, 51.02%), *AdMLP* (28, 28.57%), and *AiMLP* (20, 20.41%). ARE elements existed in 103 peanut *MLP* genes, including *AhMLP* (51, 49.51%), *AdMLP* (29, 28.16%), and *AiMLP* (23, 22.33%). Therefore, it was concluded that peanut *MLP* genes were mostly regulated by transcription factors and plant hormones-responsive elements.

**Table 1:** Number of Cis acting elements in peanut *MLP* genes.

Cis acting element	Gene	Number (Ratio)
MYB	*AhMLP*	67(51.54%)
	*AdMLP*	35(26.92%)
	*AiMLP*	28(21.54%)
	total	130
ERE	*AhMLP*	62(51.67%)
	*AdMLP*	30(25.00%)
	*AiMLP*	28(23.33%)
	total	120
MYC	*AhMLP*	62(52.10%)
	*AdMLP*	33(27.73%)
	*AiMLP*	24(20.17%)
	total	119
ABRE	*AhMLP*	50(51.02%)
	*AdMLP*	28(28.57%)
	*AiMLP*	20(20.41%)
	total	98
ARE	*AhMLP*	51(49.51%)
	*AdMLP*	29(28.16%)
	*AiMLP*	23(22.33%)
	total	103

### 3.6 Expression analysis of *AhMLP* genes under different abiotic stresses by RNA-seq and qRT-PCR

**Figure 5:**
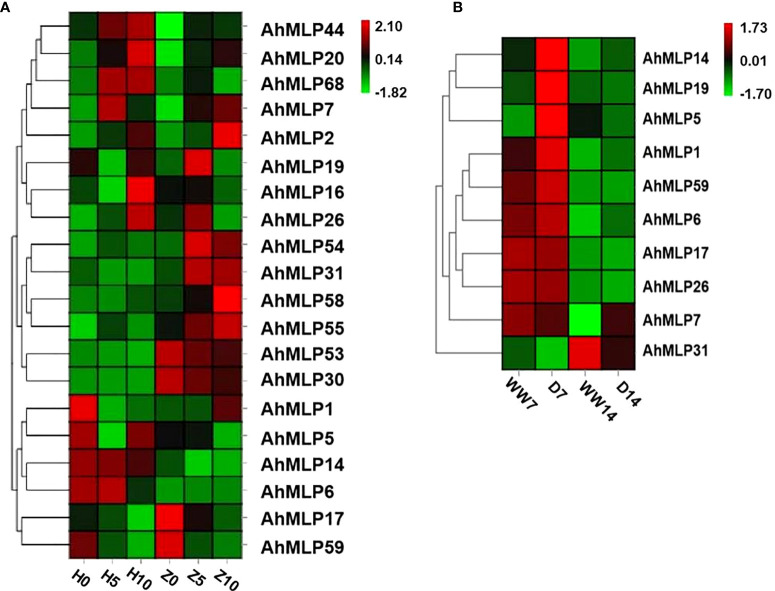
*AhMLP* genes expression level under Waterlogging stress and drought stress by RNA-seq. **(A)** Gene expression level under waterlogging stress. H0, H5 and H10 represent HuaYu39 waterlogging treatment for 0, 5 and 10 days; Z0, Z5 and Z10 represent Zhongkaihua 1 waterlogging treatment for 0, 5 and 10 days **(B)** Gene expression level under drought stress. WW7 and WW14 represent HuaYu39 treated after 7 and 14 days under well-water; D7 and D14 represent HuaYu39 drought treatment for 7 and 14 days. The heatmap was generated by Omicsmart website (https://www.omicsmart.com/home.html/), and the fragments per kilobase of transcript per million fragments (FPKM) values of peanut *MLP* genes were log2-transformed. The red and blue colors represent the maximum and minimum values, respectively.

To explore the expression pattern of *AhMLP* genes under abiotic stress, heat map analyses were performed based on the transcriptome data measured in our previous research (Huang et al., 2020; Zeng et al., 2021). Under waterlogging stress, the expression level of 20 *AhMLP* genes was significantly different during waterlogging stress treatment for 0, 5 and 10 days between waterlogging-sensitive varieties HY39 and waterlogging-resistant varieties ZKH1 (**Figure 5A**, **Table S7**). The expression level of *AhMLP54*, *AhMLP31*, *AhMLP58* and *AhMLP55* were significantly increased after 5 and 10 days in ZKH1, but which were lower in HY39. Under drought stress, the expression level of 10 *AhMLP* genes was significantly different during drought stress treatment for 7 and 14 days. The expression levels of *AhMLP14, AhMLP19* and *AhMLP5* genes were higher under drought treatment for 7 days (D7) than that under well-watered condition (WW) (**Figure 5B**, **Table S8**). Compared with D7 stage, *AhMLP31* expression was up-regulated in D14 stage. It was concluded that *AhMLP31* was involved in both waterlogging and drought stress.

**Figure 6:**
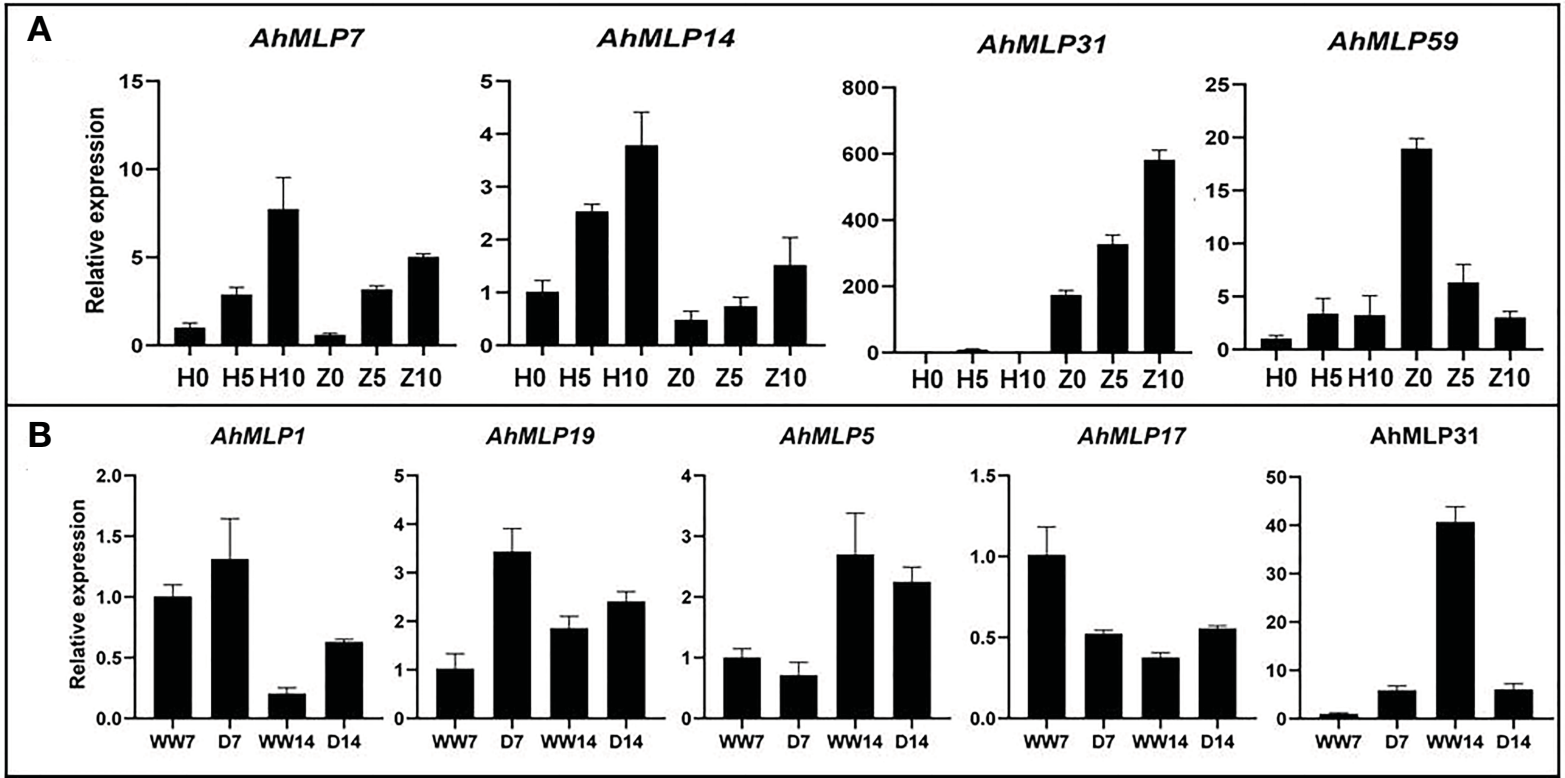
Genes expression of selected *AhMLPs* under Waterlogging stress and drought stress by qRT-PCR. **(A)** Gene expression level of ten selected *AhMLPs* under waterlogging stress. **(B)** Gene expression level of eight selected *AhMLPs* under drought stress. The data are presented as the mean ± SD (n=3), and the values differed significantly at P < 0.05. Different letters indicate significant differences.

To verify the expression data obtained by RNA-seq, qRT-PCR was used to examine the expression pattern of *AhMLP* genes under waterlogging and drought stress (**Figure 6**). The expression level of four and five *AhMLP* genes under waterlogging and drought stress was consistent with the transcriptome data. Among of them, *AhMLP31* was differently expressed under both two stresses. For waterlogging stress, *AhMLP31* was up-regulated in waterlogging-resistant ZKH1. And for drought stress, it was down-regulated at 14 days under drought stress.

### 3.7 Expression profile of *AhMLP* genes in different peanut tissues

**Figure 7:**
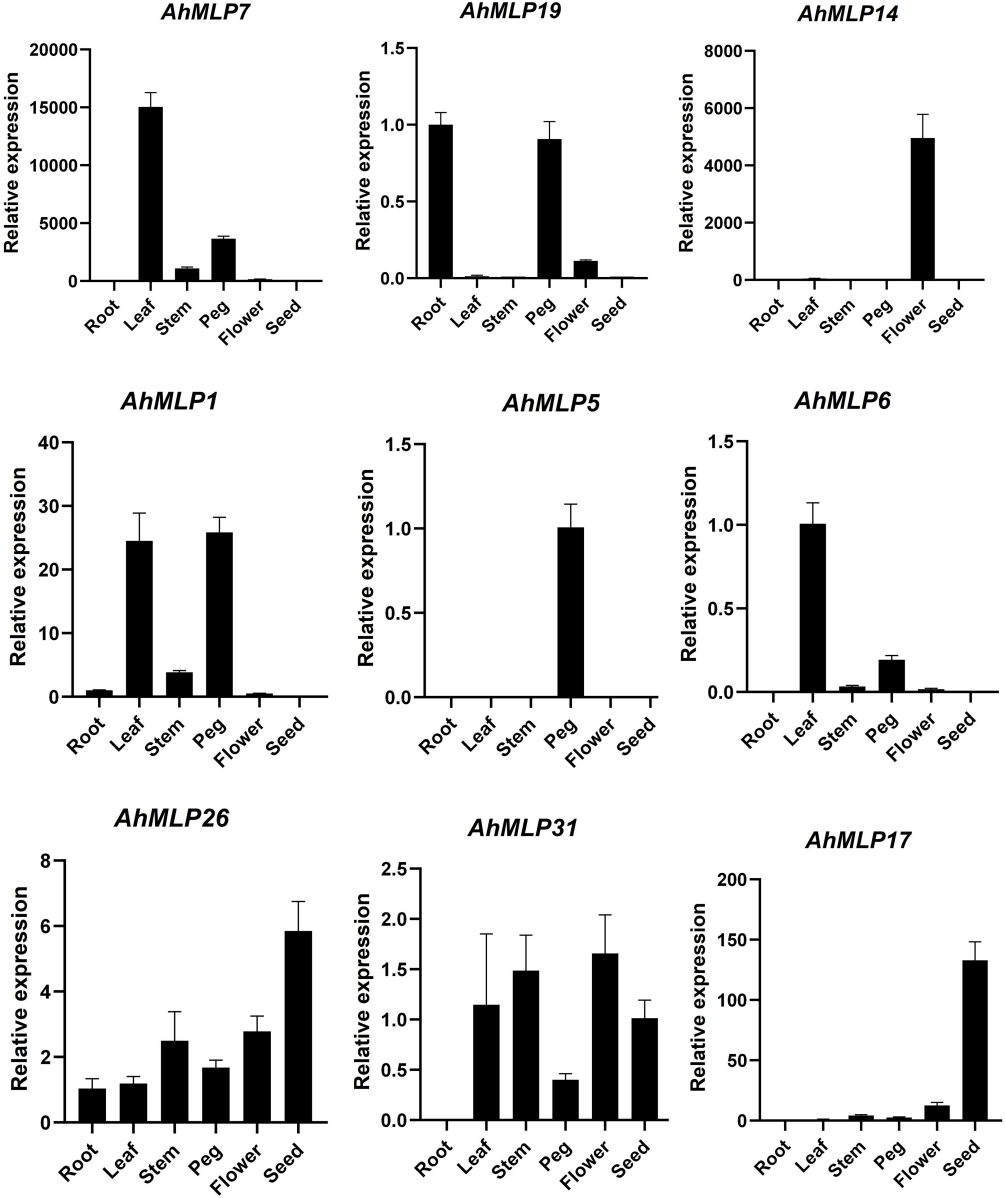
The expression pattern of *AhMLP* genes in different tissues.

The tissue-specific expression profile of *MLP* genes were analyzed by qRT-RCR in six tissues, including roots, stems, leaves, flowers, pegs and seeds from peanut (**Figure 7**, **Table S9**). Some *MLP* genes displayed tissue-specific or preferential expression patterns. For example, *AhMLP14*, *AhMLP5*, *AhMLP6* and *AhMLP17* were only differently expressed in flowers, pegs, leaves and seeds, respectively. *AhMLP7* was significantly expressed in pegs, especially leaves. *AhMLP1* was significantly expressed in leaves and pegs. Moreover, *AhMLP26* and *AhMLP31* were constitutively expressed in most of tissues.

## 4 Discussion

*MLP* genes play an important role in plant growth and development (Carella et al., 2016), biotic and abiotic stress (Chen and Dai, 2010; Yang et al., 2015; He et al., 2020). They have been identified in several plant species (Chen and Dai, 2010; Yang et al., 2015; Zhang et al., 2018; He et al., 2020). The publication of the peanut genome provided the opportunity to study the characteristics of the MLP family in peanut (Bertioli et al., 2011). In this study, 135 *MLP* genes were identified from peanut. Totally, the physical and chemical properties, phylogenetic tree, gene structure, conserved motifs, and promoter cis-acting elements were analyzed using bioinformatics methods. Moreover, the polygenetic analysis, chromosomal locations and syntenic analysis, expression profile of *MLP* genes under drought and waterlogging stresses were systematically investigated.

Gene duplication is thought to have contributed much to the evolution of morphological and physiological diversity in plants (Qiao et al., 2019). In our study, chromosomal location analysis showed that *AhMLPs* were located on Chr03, Chr05, Chr07, Chr08, Chr09 and Chr10 of sub-genome AA and BB. Moreover, *AdMLPs* were located on ChrA03, ChrA05, ChrA07, ChrA08, ChrA09 and ChrA10. And *AiMLPs* were located on ChrB03, ChrB05, ChrB07, ChrB08, ChrB09 and ChrB10. This was consistent to that the perfect synteny between AA genome and BB genome from *A. duranensis* and *A. ipaensis* (Bertioli et al., 2016). In addition, we also found that tandem and fragmental duplication were appeared on the end of the chromosome. Thus, we speculated gene duplication at the end of chromosomes led to generate multi-functions of members of gene family to enhance the ability of plant to adapt to the environment.

Cis-acting promoter elements are involved in the regulation of gene expression by the interaction between promoter bind sites and transcription factors (Singh et al., 2002). Our results showed that 135 *MLP* genes contained several functional elements, such as MYB, MYC, ERE, ABRE and ABE elements. Among these elements, the number of MYB, ERE, ABRE and ABE element was the largest, and presented in most of peanut *MLP* genes. MYB could promote the induction of various developmental and stress response genes, thus play an important role in enhancing plant tolerance to a variety of abiotic stresses (Li et al., 2021). Moreover, ERE element was presented in 62 *AhMLP* genes, which was considered as a target of ABA or ethylene signal transduction and plays an important role in ethylene and ABA regulation in plants (Ohme-Takagi and Shinshi, 1995; Wu et al., 2018). These results were consistent with the analysis of cis-acting elements in crops such as apple (Yuan et al., 2020), and indicated that *MLP* genes may play an important role in the regulation of adaptation to environmental stress and growth (Nakashima et al., 2009; Yoshida et al., 2014; Pan et al., 2020).

Drought and waterlogging are the key stresses that have a negative impact on crop yield (Dietz et al., 2021; Tong et al., 2021). The expression characteristics of *AhMLP* genes under drought and waterlogging were analyzed by RNA-seq and qRT-PCR. Among these *AhMLP* genes, the expression changes of *AhMLP31* under waterlogging stress were consistent between transcriptome and qRT-PCR. The expression of *AhMLP31* in waterlogging-resistant variety ZKH1 was upregulated and reached the peak at 10 days after waterlogging, whereas it was not detected in waterlogging-susceptible variety HY39. Further investigation was needed to confirm the roles of *AhMLP31* in the response to waterlogging stress. The MLPs, PR-10s and Bet v 1 proteins share a common fold characterized by a solvent-accessible hydrophobic cavity, which serves as a binding site for small-molecule ligands, mostly hormones and flavonoids (Radauer et al., 2008). In cotton, GhMLP28 interacted with cotton ethylene response factor 6 (GhERF6) and facilitated the binding of GhERF6 to GCC-box element (Yang et al., 2015). Therefore, we speculate that AhMLP31 interacts with other transcription factors, contributing with resistance to waterlogging and drought in peanut. To elucidate the molecular mechanism by which AhMLP31 may act in the responses to abiotic stress, yeast two-hybrid, pull-down screening and luciferase complementation imaging (LCI) assays will further conducted to identify proteins that interact with AhMLP31.

## 5 Conclusion

In this study, 135 *MLP* genes were identified from the genome of *A. hypogaea*, and two diploid species *A. duranensis* and *A. ipaensis* and divided into five groups. The analysis of gene structures and protein motifs revealed that most *MLPs* in peanut were relatively conserved. The 135 *MLP* genes were distributed in 12 chromosomes of *A. hypogaea*, six chromosomes of *A. duranensis* and *A. ipaensis*, respectively. It was found that cis-acting regulatory elements in *MLP* gene promoters were related to abiotic/biotic. The results of expression profiles indicated that *MLP* genes were involved in response to waterlogging and drought stress in peanut. Our research results provided a basis for new insights into the biological function of *MLP* genes in plant.

## Data availability statement

The original contributions presented in the study are included in the article/**Supplementary Material**. Further inquiries can be directed to the corresponding authors.

## Author contributions

TC and LZ conceived and designed the study. JL performed the experiments and wrote the original draft. ZH and RZ participated in the bioinformatics analysis. HG and SL prepared the plant materials. YG, SY, YW and ZH performed the data analysis. TC and LZ revised the manuscript. All authors contributed to the article and approved the submitted version.
