# Supplementary figures and images for: Genome-wide characterization of major latex protein gene family in peanut and expression analyses under drought and waterlogging stress
Source: Front Plant Sci. 2023 Apr 18;14:1152824. doi: 10.3389/fpls.2023.1152824 (PMC10151671; doi:10.3389/fpls.2023.1152824)

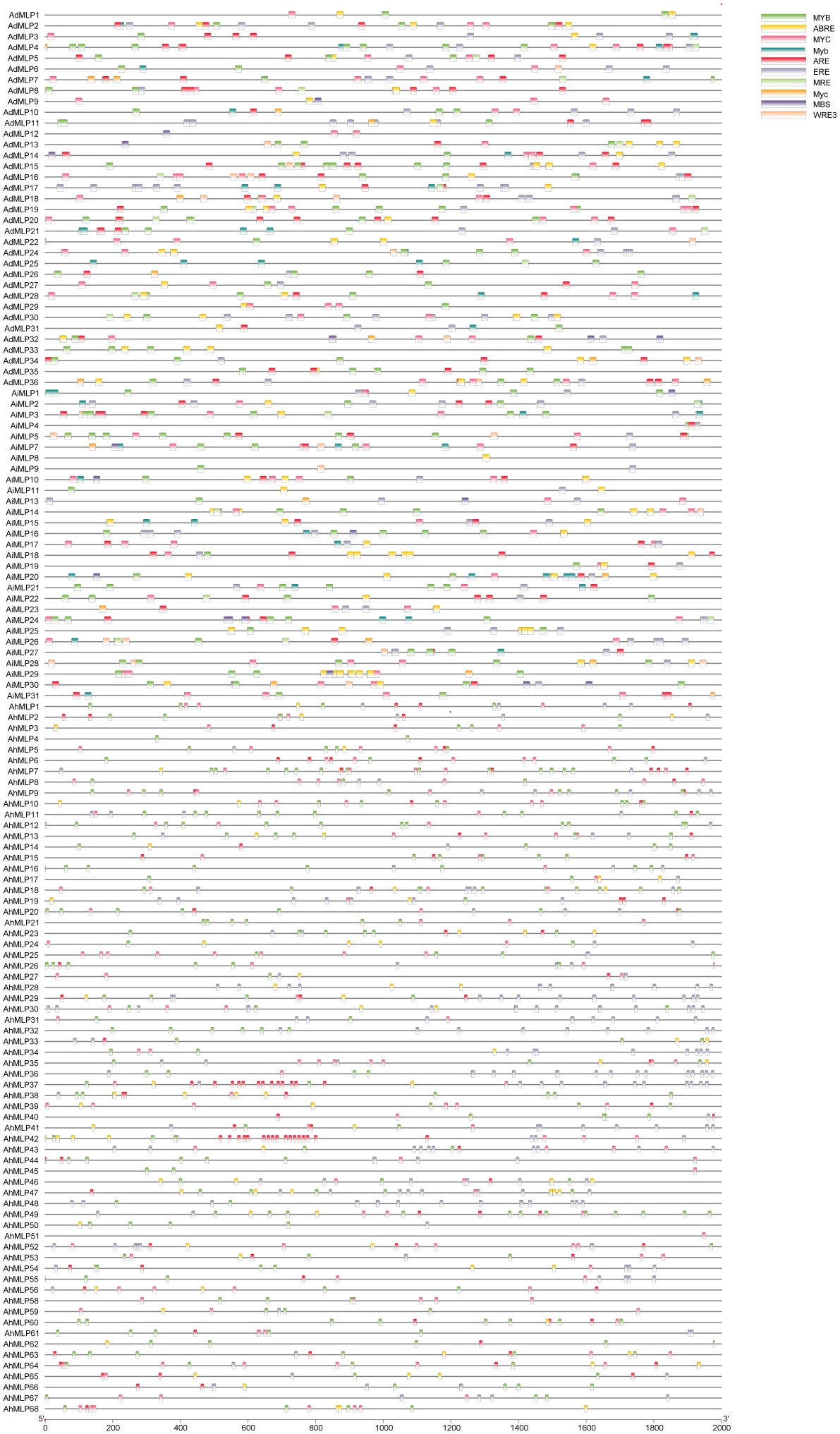

Supplement: Supplementary file 1 [file Image_1.jpeg]
